# Neurorestorative Effects of a Novel Fas-Associated Factor 1 Inhibitor in the MPTP Model: An [^18^F]FE-PE2I Positron Emission Tomography Analysis Study

**DOI:** 10.3389/fphar.2020.00953

**Published:** 2020-06-25

**Authors:** Hyun Soo Park, Yoo Sung Song, Byung Seok Moon, Sung-Eun Yoo, Jae Moon Lee, Yeon-Tae Chung, Eunhee Kim, Byung Chul Lee, Sang Eun Kim

**Affiliations:** ^1^ Department of Nuclear Medicine, Seoul National University Bundang Hospital, Seoul National University College of Medicine, Seongnam, South Korea; ^2^ Department of Transdisciplinary Studies Graduate School of Convergence Science and Technology, Seoul National University, Seoul, South Korea; ^3^ Department of Nuclear Medicine, Ewha Womans University Seoul Hospital, Ewha Womans University College of Medicine, Seoul, South Korea; ^4^ Kainos Medicine, Inc., Seongnam, South Korea; ^5^ Department of Bioscience and Biotechnology, Chungnam National University, Daejeon, South Korea; ^6^ Advanced Institutes of Convergence Technology, Suwon, South Korea

**Keywords:** Fas-associated factor 1 inhibitor, KM-819, Parkinson’s disease, non-displaceable binding potential, positron emission tomography

## Abstract

Fas-associated factor 1 (FAF1), a Fas-binding protein, is implicated in neuronal cell death in Parkinson’s disease (PD). We examined the effects of a novel FAF1 inhibitor, KM-819, in dopaminergic neurons in a 1-methyl-4-phenyl-1,2,3,6-tetrahydropyridine (MPTP) mouse model using [^18^F]FE-PE2I positron emission tomography (PET). The MPTP model was generated with subacute MPTP treatment (20 mg/kg/day, i.p.) for 5 consecutive days in C57bl/6J mice. This study included three groups: the control group (treatment with saline only), the MPTP model group with KM-819 treatment (20 mg/kg/day p.o.) for 6 days, and the MPTP model group without KM-819 treatment. [^18^F]FE-PE2I PET studies were conducted in the same animals before and after MPTP with or without KM-819 treatment to monitor changes in striatal dopamine transporter activity indicated by non-displaceable binding potential (BP_ND_) of [^18^F]FE-PE2I, and the expression levels of tyrosine hydroxylase were assessed using immunohistochemistry before and after KM-819 treatment. After MPTP injection, decreased striatal BP_ND_ was observed in the MPTP model group compared with the control group. Striatal BP_ND_ increased in the MPTP model group with KM-819 treatment, but not in the MPTP model group without KM-819 treatment. The tyrosine hydroxylase expression levels also significantly increased in the MPTP model group with KM-819 treatment compared with the control group. This study indicates that inhibition of the Fas-mediated cell death pathway by KM-819 has neurorestorative effects in striatal dopamine neurons in the MPTP model. Further studies would be needed to investigate the potential of KM-819 as a therapeutic drug for PD treatment.

## Introduction

Parkinson’s disease (PD) is a progressive neurodegenerative disorder that mainly affects movement, resulting in various motor-related symptoms such as tremor, rigidity, and bradykinesia (Dauer and Przedborski, 2003). Accumulation of alpha-synuclein in the form of Lewy bodies is the main known pathology and causes neuronal degeneration of the dopaminergic innervation in the nigrostriatal pathway. In addition, several other mechanisms have been suggested to be involved in neuronal cell death during the development and progression of PD (Lev et al., 2003; Tatton et al., 2003). It is currently accepted that neuronal death may be attributed to oxidation of dopamine, apoptosis, impaired autophagy, neuroinflammation, and disruption of calcium homeostasis (Zeng et al., 2018). However, little is known about molecular mechanisms by which the signal transduction pathways induce neuronal cell death.

Levodopa remains as the main treatment option for PD; however, levodopa does not reverse or delay the progression of neurodegeneration in PD (Schrag et al., 2000). Moreover, the effect of levodopa decreases over time, and levodopa eventually induces side effects such as dyskinesia and motor fluctuations (Bastide et al., 2015). Therefore, numerous efforts have been made to develop other types of disease-modifying drugs that could change the paradigm of PD treatment.

Fas protein, a member of the tumor necrosis factor receptor family, interacts with Fas ligand and induces apoptosis (Yu and Shi, 2008; Menges et al., 2009). Fas-associated protein 1 (FAF1), which was first identified as an intracellular domain binding protein of Fas, potentiates Fas-mediated apoptosis (Chu et al., 1995). Several binding partners and protein interactions of FAF1 have been verified, and Fas-associated protein with death domain, caspase-8, protein kinase CK2 (formerly termed casein kinase II), and ubiquitin proteins are known FAF1 interacting proteins that regulate apoptosis (Guerra et al., 2001; Ryu et al., 2003; Song et al., 2005). In addition, the role of FAF1 in the pathophysiology of several neurodegenerative diseases such as PD has brought the attention. In PD, FAF1 may be an integral component of progressive neurodegeneration of the dopaminergic innervation. In particular, FAF1 is a substrate of parkin, a ubiquitin E3 ligase. Inactivation of parkin induces the overexpression of FAF1, thus resulting in increased dopaminergic neuronal cell death (Sul et al., 2013). Notably, in human brain postmortem tissues of PD patients, FAF1 expression levels significantly increase in the frontal cortex, and the distribution is associated with the accumulation of Lewy bodies and the decrease of dopamine transporter expression (Betarbet et al., 2008). Accordingly, FAF1 has recently gained attention as a new therapeutic target. Preclinical studies have suggested that a FAF-1 inhibiting molecule KM-819, first developed by Kainos Medicine, Inc. (Seongnam, Korea), slows down the progression of PD by inhibiting the Fas-mediated apoptosis (Shin et al., 2019). The drug has undergone a phase I clinical trial in Korea, and has shown favorable safety, tolerability, phamacokinetic, and phamacodynamic results (Shin et al., 2019). [^18^F]FE-PE2I is a well-known radioligand for the assessment of dopaminergic innervation, which has its strength in quantifying dopamine transporter expression(Sasaki et al., 2012). In this study, we evaluated *in vivo* effects of the novel FAF1 inhibitor, KM-819, in a subacute 1-methyl-4-phenyl-1,2,3,6-tetrahydropyridine (MPTP) mouse model of PD using [^18^F]FE-PE2I positron emission tomography (PET).

## Materials and Methods

### Animals

Male C57bl/6J mice (age, 8 weeks; body weight, 22.4 ± 0.8 g) were randomly assigned to three groups (*n* = 30, 10 per group): the control group, the MPTP model group without KM-819 treatment, and the MPTP model group with KM-819 treatment. In the MPTP model group, mice were injected with MPTP (15 mg/kg; Sigma-Aldrich, St. Louis, MO, USA) intraperitoneally for 5 consecutive days. For KM-819 treatment, KM-819 (Kainos Medicine, Inc., Seongnam, Korea) was dissolved in 0.5% methylcellulose and was orally administered (20 mg/kg) for 6 consecutive days, starting from 48 h after the last dose of MPTP. Animals underwent 60 min dynamic 2-[^18^F]fluoroethyl 8-[(2E)-3-iodoprop-2-en-1-yl]-3-(4-methyl phenyl)-8-azabicyclo[3.2.1]octane-2-carboxylate ([^18^F]FE-PE2I) brain PET/CT scans on day 0 (baseline; scan 1), day 6 (scan 2), and day 13 (scan 3) (**Table 1**). This study was approved by the Institutional Animal Care and Use Committee of the Seoul National University Bundang Hospital.

**Table 1:** Schedule and dosing regimen.

Groups	Day 0	Day 1 - Day 5	Day 6	Day 7- Day 12	Day 13
**Control** **(*n* = 10)**	[^18^F]FE-PE2I PET/CT scan **(scan 1)**	Normal saline, *i.p.*	[^18^F]FE-PE2I PET/CT scan **(scan 2)**	Normal saline, *p.o.*	[^18^F]FE-PE2I PET/CT scan **(scan 3)**
**MPTP model without KM-819 (*n* = 10)**		MPTP, 15 mg/kg, *i.p.*, 5 days		Normal saline, *p.o.*	
**MPTP model with KM-819 (*n* = 10)**		MPTP, 15 mg/kg, *i.p.*, 5 days		KM-819, 20 mg/kg, *p.o.*, 6 days	

### [^18^F]FE-PE2I PET Imaging and Analysis

[^18^F]FE-PE2I was synthesized from the tosylate-precursor by Kryptofix-mediated nucleophilic aliphatic ^18^F-substitution in a TRACERlab FX N Pro chemistry synthesizer (GE Healthcare, Milwaukee, USA) according to previously published in literature (Bang et al., 2016). The radioactivity yield was 17.9 ± 3.4% (n = 22, non-decay corrected) with over 98% radiochemical purity. The molar activity was 295 ± 64 GBq/μmol at the time of end-of-synthesis (EOS). Before imaging, mice were anesthetized with 1.5–2% isoflurane (2 L/min flow rate). Animals underwent 60 min dynamic [^18^F]FE-PE2I brain PET/CT scans according to the schedule. PET/CT imaging was performed in a dedicated small-animal PET/CT (NanoPET/CT, Mediso Ltd., Budapest, Hungary). Axial field of view (FOV) was 10 cm, transaxial FOV was 12 cm, and the spatial resolution of PET was 1.2 mm full-width at half-maximum (FWHM) at the center of FOV. Attenuation correction was performed with CT scans, and PET acquisition was concomitantly started after 6.9 ± 0.9 MBq of [^18^F]FE-PE2I injection (10.4 ± 1.4 ng of FE-PE2I at time of injection). [^18^F]FE-PE2I was injected intravenously *via* the tail vein at a volume of 200 μL. Dynamic PET images were acquired for 60 min at 48 frames (12 frames × 10 s, 16 frames × 30 s, 8 frames × 1 min, and 10 frames × 4 mins). PET images were reconstructed with the 3D adjoint Monte Carlo method, with scatter and random corrections, and voxel dimensions were 0.6×0.6×0.6 mm^3^. Counts of each frame were corrected for radioactive decay to the time of injection. Images were analyzed after spatial normalization to the predefined magnetic resonance imaging (MRI) mouse brain template using the PMOD software (PMOD Technologies, Switzerland). Time-activity curves were measured in the striatum, midbrain, and cerebellum, and non-displaceable binding potential (BP_ND_) of [^18^F]FE-PE2I estimation and BP_ND_ parametric image generation were performed using a simplified reference tissue model (Ichise’s multilinear reference tissue model) with the cerebellum as the reference tissue (Ichise et al., 2003).

### Immunohistochemistry

After [^18^F]FE-PE2I PET/CT imaging, under anesthesia with isoflurane, the mice were transcardiacally perfused with PBS, followed by 4% paraformaldehyde. The brains were removed and post-fixed in 4% paraformaldehyde for 6 h and were then immersed in 20% sucrose overnight at 4°C prior to sectioning on a microtome-cryostat at a thickness of 40 μm (Microm HM 550 MP, UK). Six sections were obtained from each examined brain region. The free-floating sections were washed briefly with PBS three times, and the endogenous peroxidase activity was then suppressed by incubation with 3% H_2_O_2_. The sections were preincubated with 3% bovine serum albumin (BSA) and 0.1% Triton 100 in PBS for 30 mins and were then incubated with anti-tyrosine hydroxylase antibodies (Millipore, USA, 1: 5,000) in PBS containing 0.3% BSA and Triton X-100. After overnight incubation at 4°C, the sections were incubated in biotinylated anti-mouse immunoglobulin G (Vector Laboratories, USA, 1:200) in PBS containing 0.3% BSA and Triton X-100 for 1 h. The sections were subsequently incubated for 1 h at room temperature with streptavidin-biotin-horseradish peroxidase complex (Vectastain, ABC kitElite, Vector Laboratories). The TH immunoreactivity was visualized using 3,3-diaminobenzidine (DAB). The sections were then mounted on glass slides, dehydrated, and imaged under a light microscope (Axio Observer, Zeiss, Germany) with Image-Pro Plus 7.0 software (MediaCybernetics, Silver Spring, MD). Quantitative analysis of the TH expression was assessed by computer densitometry, from the digital images captured with the light microscope. The color deconvolution ImageJ software was used to evaluate the percentage of TH expression.

### Statistical Analysis

ANOVA followed by Scheffe’s test was used to test the statistical significance of within-subject changes and group mean differences in [^18^F]FE-PE2I BP_ND_. Kolmogorov–Smirnov test was performed to assess normal distribution. Within-group differences were compared using repeated ANOVA for parametric variables and Friedman test for nonparametric variables. A *P*-value of < 0.05 was considered statistically significant. Statistical analysis was conducted with Prism software (version 5; GraphPad Software, Inc., CA, USA).

## Results

### Changes in Striatal Dopaminergic Transporter Activity

**Figure 1:**
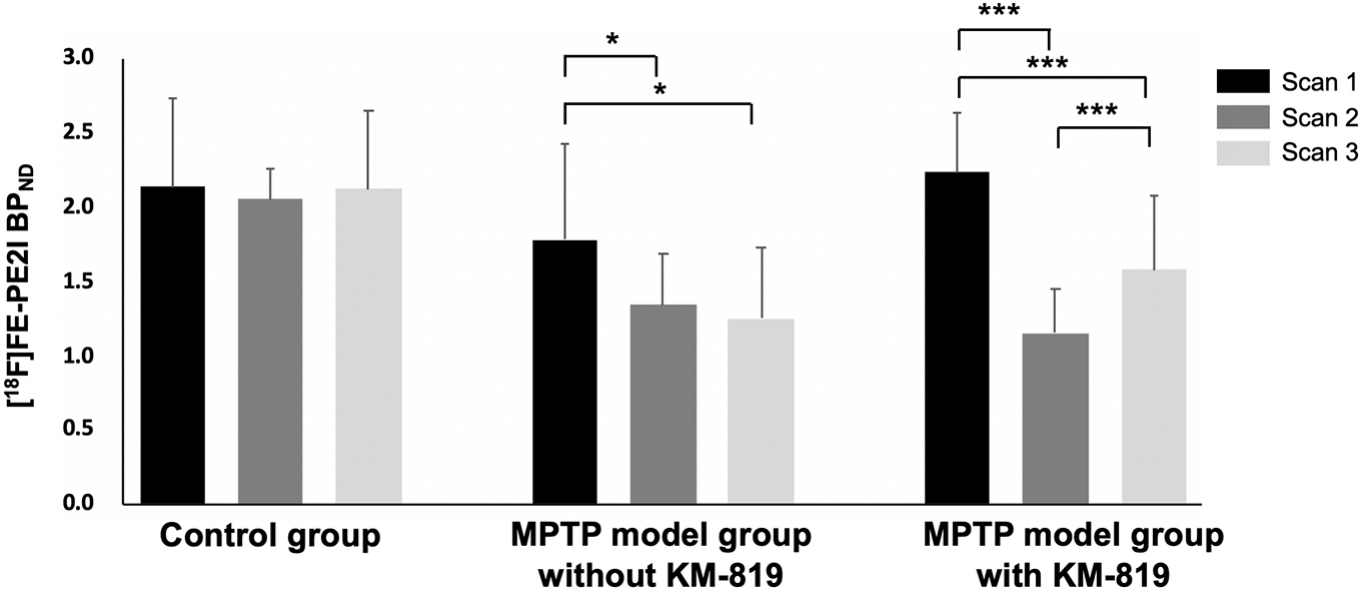
Changes in striatal [^18^F]FE-PE2I BP_ND_ in the control group, the MPTP model group without KM-819 treatment, and the MPTP model group with KM-819 treatment. Error bars indicate standard deviation. (^*^ *P* < 0.05, ^***^ *P* < 0.001).

**Figure 2:**
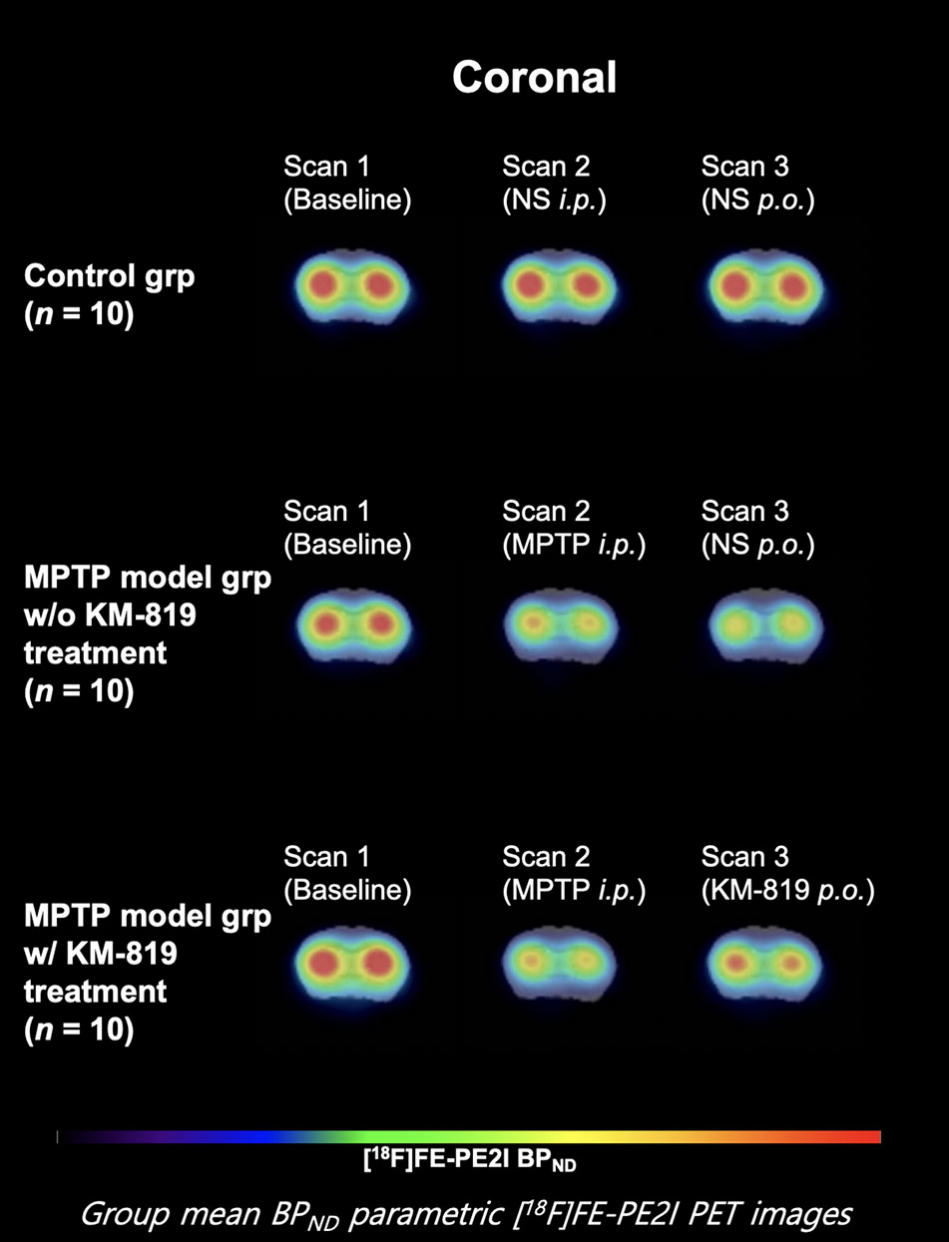
Mean striatal BP_ND_ parametric [^18^F]FE-PE2I PET images at each scan in each group. Coronal images 1.0 mm anterior from the bregma.

**Figure 1** shows the striatal [^18^F]FE-PE2I BP_ND_ at scan 1, scan 2, and scan 3 in each group. The PD mouse model was successfully generated by subacute MPTP treatment, as indicated by substantially reduced striatal dopaminergic transporter activity at scan 2 in the MPTP model group compared with the control group (**Figure 1**, **Table 2**). Significant increases in striatal [^18^F]FE-PE2I BP_ND_ were observed at scan 3 in the MPTP model group with KM-819 injection, but not in the MPTP model group without KM-819 injection. However, the striatal [^18^F]FE-PE2I BP_ND_ at scan 3 in the MPTP model group with KM-819 injection did not recover to the baseline (scan 1) level. Mean BP_ND_ parametric [^18^F]FE-PE2I PET images at each scan in each group are shown in **Figure 2**.

**Table 2:** Striatal [^18^F]FE-PE2I BP_ND_.

Groups	Scan 1	Scan 2	Scan 3
**Control**	2.1 ± 0.6	2.1 ± 0.2	2.1 ± 0.5
**MPTP model without KM-819**	1.8 ± 0.7	1.3 ± 0.3	1.2 ± 0.5
**MPTP model with KM-819**	2.2 ± 0.4	1.2 ± 0.3	1.6 ± 0.5

### Changes in Midbrain Dopaminergic Transporter Activity

**Figure 3:**
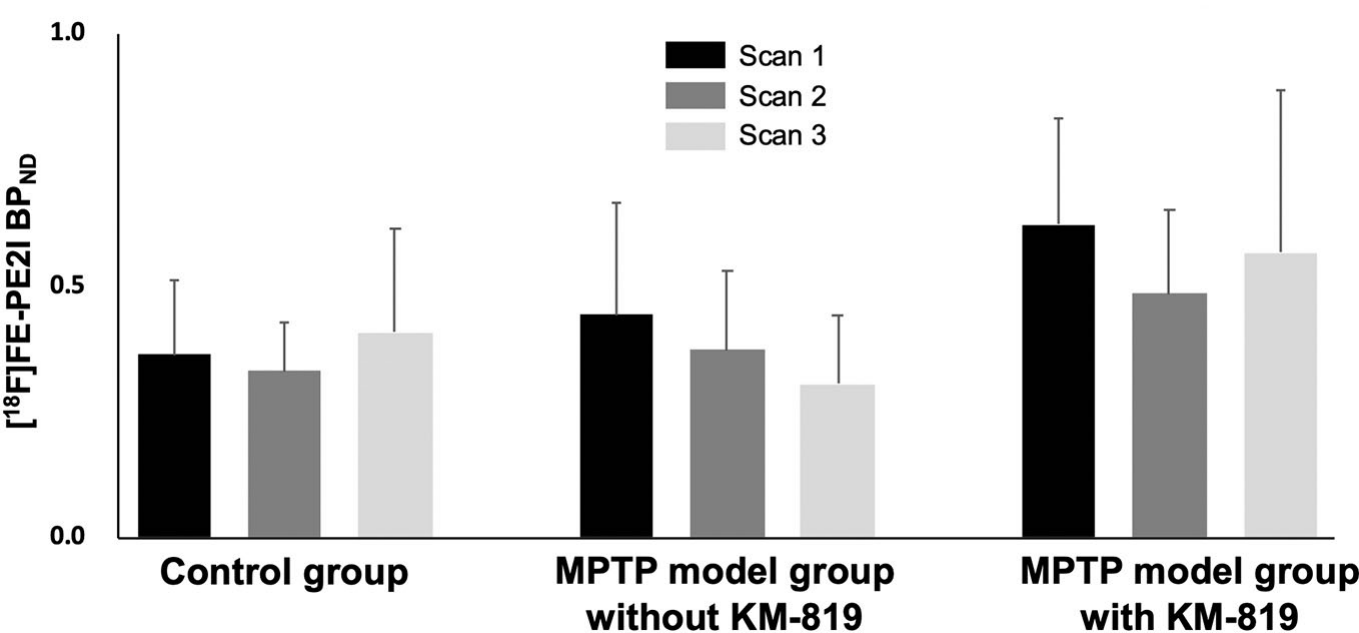
Changes in midbrain [^18^F]FE-PE2I BP_ND_ in the control group, the MPTP model group without KM-819 treatment, and the MPTP model group with KM-819 treatment. Error bars indicate standard deviation.

**Figure 4:**
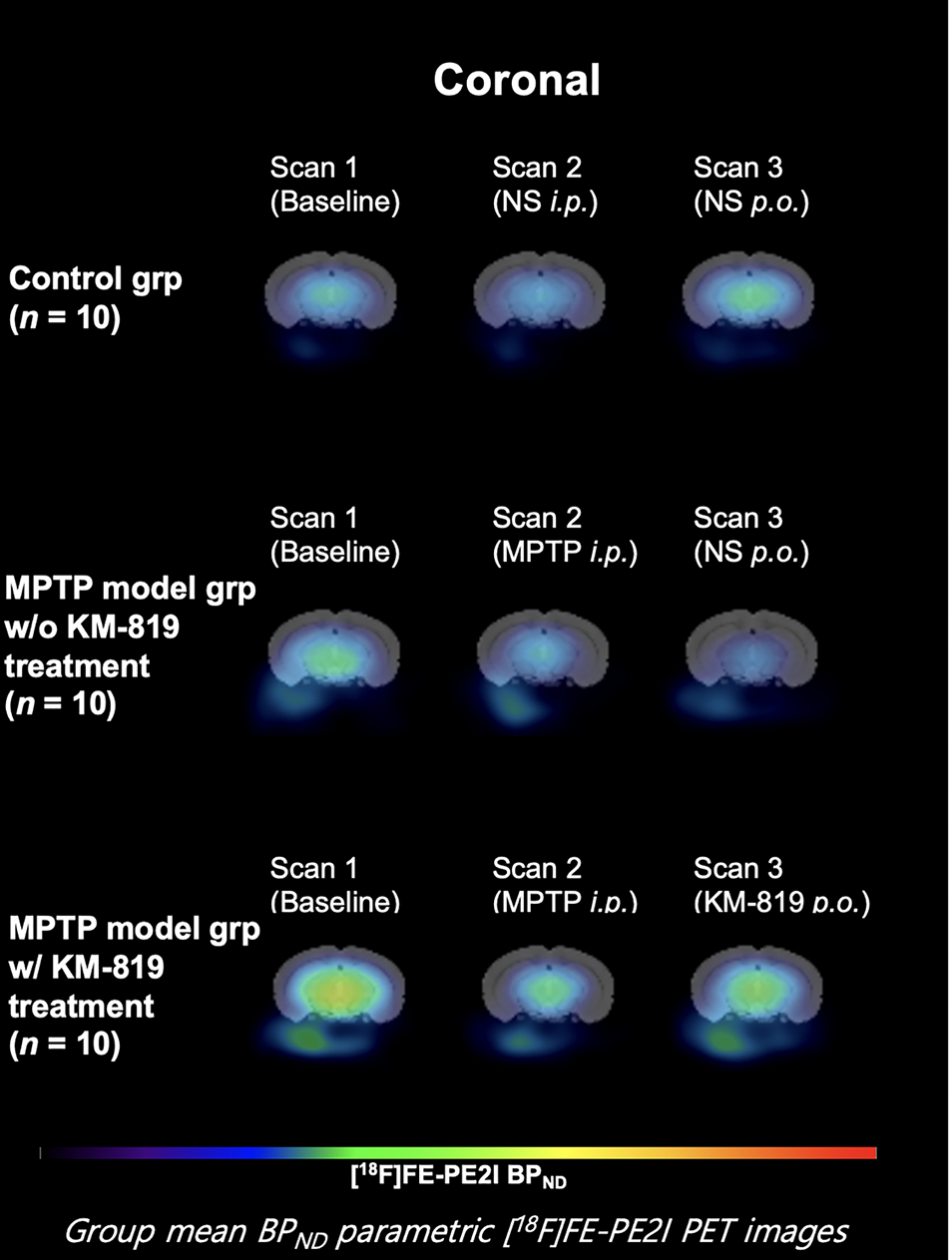
Mean midbrain BP_ND_ parametric [^18^F]FE-PE2I PET images at each scan in each group. Coronal images 6.8 mm posterior from the bregma.

**Figure 3** and **Table 3** show midbrain BP_ND_ at scan 1, scan 2, and scan 3 in each group. No statistically significant differences in mean BP_ND_ were found between groups or scans. Mean BP_ND_ parametric [^18^F]FE-PE2I PET images at each scan in these groups are shown in **Figure 4**.

**Table 3:** Midbrain [^18^F]FE-PE2I BP_ND_.

Groups	Scan 1	Scan 2	Scan 3
**Control**	0.4 ± 0.1	0.3 ± 0.1	0.4 ± 0.2
**MPTP model without KM-819**	0.4 ± 0.2	0.4 ± 0.2	0.3 ± 0.1
**MPTP model with KM-819**	0.6 ± 0.2	0.5 ± 0.2	0.6 ± 0.3

### Histopathology

**Figure 5:**
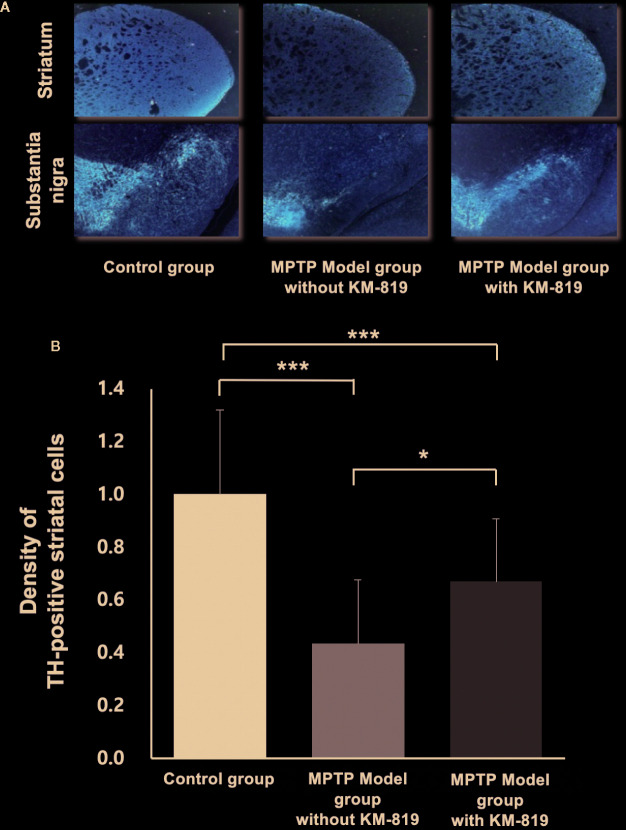
Immunohistochemical analysis of tyrosine hydroxylase. **(A)** Representative tyrosine hydroxylase immunohistochemistry in the control group, the MPTP model group without KM-819 treatment, and the MPTP model group with KM-819 treatment. **(B)** Quantitative comparison of the tyrosine hydroxylase expression levels among the control group, the MPTP model group without KM-819 treatment (saline), the MPTP model group with levodopa treatment, and the MPTP model group with KM-819 treatment. Error bars indicate standard deviation. (^*^ *P* < 0.05, ^***^ *P* < 0.001).

Tyrosine hydroxylase was assessed using immunohistochemistry to investigate the effect of KM-819 (**Figure 5**). Significant decreases in striatal tyrosine hydroxylase expressions were observed in the MPTP model group with saline injection compared with the control group. However, significant increases in tyrosine hydroxylase expressions were found in the MPTP model group with KM-819 injection compared with the MPTP model with levodopa or saline injection group.

## Discussion

Levodopa does not delay the progression of PD and has limited cumulative dosages that can be used over the course of the disease. Therefore, researchers have put their efforts in developing novel therapeutic agents. Recent strategies for PD drug development have focused on discovering novel targets that may have the potential to modify and slow the progression of the disease. KM-819, which was first developed as a non-dopaminergic therapeutic agent, currently requires further evaluation to prove its effects on dopaminergic innervation (Shin et al., 2019).

Parkin, an E3 ubiquitin ligase, is known to play a critical role in ubiquitination. In normal conditions, parkin inhibits the apoptotic effect of FAF1 by promoting its degradation (Sul et al., 2013). Notably, in some PD patients, mutations of the *parkin* gene have been identified and are known to be associated with dopaminergic neuronal cell loss in the substantia nigra and the locus caeruleus (Mori et al., 1998). Recent clinical studies indicate that mutations in the *parkin* gene contribute to the development of early-onset PD (Lucking et al., 2000; Clark et al., 2006). In our study, histopathological analyses revealed that decreased tyrosine hydroxylase expressions were attenuated in the striatum in the MPTP model with KM-819 treatment, suggesting that KM-819 may have the potential to restore dopaminergic innervation.

In our study, decreased striatal BP_ND_ at scan 2 (-48% compared with baseline) improved at scan 3 (-29% compared with baseline) in the MPTP model with KM-819 treatment. [^18^F]FE-PE2I is known for its suitable affinity to dopaminergic transporters, although it has low affinity to other monoaminergic transporters (Schou et al., 2009). In a previous clinical study with [^18^F]FE-PE2I PET, quantitative analysis of striatal [^18^F]FE-PE2I BP_ND_ showed a positive correlation between the disease duration and Unified Parkinson’s Disease Rating Scale motor scores (Fazio et al., 2018; Li et al., 2018). The findings suggest that KM-819 may have the potential to attenuate PD-related symptoms and decrease the progression rate, though further evaluations are needed before entering clinical trials for drug efficacy. To date, although numerous studies have sought to develop disease-modifying drugs, no candidate drugs have been successful in phase III clinical studies (Olanow et al., 2011; Kieburtz and Olanow, 2015; Charvin et al., 2018), and these failures prevent the further clinical application of these drugs. For instance, gene therapy needs to be delivered by a surgical injection in the patient; however, it is unsuitable in patients with early or prodromal stage of PD. α-synuclein-targeted therapies use antibody-based drugs, but it is challenging to deliver sufficient dosages to optimal brain targets. Iron chelators and *LRRK2* inhibitors have shown adverse effects on the hematological, pulmonary, and renal systems (Charvin et al., 2018). Notably, KM-819 has just entered the phase I clinical trial, and it has high feasibility for target delivery due to its small molecule size and the low possibility of subacute toxicities (Shin et al., 2019).

A previous study demonstrated increased FAF1 expressions and decreased tyrosine hydroxylase expressions in the substantia nigra in the MPTP model (Sul et al., 2013). In our study, [^18^F]FE-PE2I PET images revealed no differences in midbrain BP_ND_. In addition, no significant improvement in tyrosine hydroxylase activity was observed in the substantia nigra compared with the striatum. Two possible reasons may account for these findings. First, the relative FE-PE2I BP_ND_ ratio is lower in the midbrain than in the striatum (ratio of 1: 5) (Jucaite et al., 2006). The role of dopaminergic transporter in the midbrain is yet to be examined. Second, FAF1-mediated neuronal cell death is ameliorated by parkin (Sul et al., 2013). However, dopaminergic cells in the substantia nigra exhibit high levels of parkin mRNA but no parkin protein; in contrast, the striatum has high levels of parkin protein (Stichel et al., 2000). Considering the lack of parkin protein in the midbrain, our findings indicate that parkin protein may be a prerequisite for the effect of KM-819 treatment on FAF1.

Our study has several limitations. First, the MPTP model has limitations in reproducing the pathophysiology of PD, in that they do not fully reproduce the movement-related phenotypes of PD (Potashkin et al., 2010; Zhang et al., 2017). Second, the degree and recovery of dopaminergic denervation is dependent on the MPTP injection protocol and mice selection, which may be a confounding factor in interpreting study results. We adopted an injection protocol with a reference that would deplete approximately 40–60% of dopaminergic neurons (Mitsumoto et al., 1998; Jackson-Lewis and Przedborski, 2007; Kuroiwa et al., 2010), with C57bl/6J mice of 8 weeks of age. Since the MPTP model is basically an acute model regardless of the previously suggested protocols, the effect of KM-819 must be interpreted carefully. Further studies are needed with older mice and mixed sex pairs. Finally, the acute treatment of KM-819 may be a confounding factor in the interpretation, since MPTP has complex toxicokinetics. Its metabolite MPP+ (1-methyl-4-phenylpyridinium), which has a high affinity for dopamine transporters, is suggested to have neurotoxicity on dopaminergic neurons by selective uptake into synaptic vesicles or by impairing the mitochondrial transport (Del Zompo et al., 1993; Kim-Han et al., 2011). Therefore, the positive effect of KM-819 may be due to its interference with the toxicokinetics of MPTP. To minimize any possible interference, we delayed the administration of KM-819 for 48 h after the last administration of MPTP (Jackson-Lewis and Przedborski, 2007), but requires further evaluation. In conclusion, our study proposes a neurorestorative effect of KM-819 on the dopaminergic innervation. The effects were not yet proven to be neuroprotective due to the described limitations, therefore further investigations would be needed to bring the investigation of KM-819 to a clinical level. Future *in vivo* rescue studies with *Parkin^-/-^* or *Faf1^gt/gt^* mice (Sul et al., 2013) may be needed to demonstrate the specific molecular mechanisms underlying the effects of KM-819.

This study demonstrated the neurorestorative effect of KM-819, a novel FAF1 targeting drug, though there are still remaining challenges in conducting future clinical trials of KM-819. Further studies are needed to evaluate the phenotypic effects of KM-819, and the pharmacologic mechanism of KM-819 on the restoration of [^18^F]FE-PE2I uptake.

## Data Availability Statement

All datasets generated for this study are included in the article/supplementary material.

## Ethics Statement

The animal study was reviewed and approved by Institutional Animal Care and Use Committee of the Seoul National University Bundang Hospital.

## Author Contributions

Conception and design of the research: HP and YS. Acquisition of data: HP, YS, BM, Y-TC, and S-EY. Analysis and interpretation of data: HP, YS, BM, and JL. Statistical analysis: HP. Obtaining funding: EK and SK. Drafting the manuscript: HP and YS. Revision of manuscript for important intellectual content: BL and SK.

## Funding

This study was supported by the Korea Health Technology R&D Project through the Korea Health Industry Development Institute (KHIDI), funded by the Ministry of Health & Welfare, Republic of Korea (HI16C0947).

## Conflict of Interest

Authors S-EY, JL, Y-TC are employed by Kainos Medicine, Inc.

The remaining authors declare that the research was conducted in the absence of any commercial or financial relationships that could be construed as a potential conflict of interest.
